# Multifunctional Alginate Composite Fibers Based on Pre-Crosslinked Spinning Solutions

**DOI:** 10.3390/ma19101933

**Published:** 2026-05-08

**Authors:** Lingchun Liu, Hanxu Zhou, Cong Du

**Affiliations:** Shandong Key Laboratory of Renewable Membrane Materials, College of Materials Science and Engineering, Qingdao University, Qingdao 266071, China; 18354453950@163.com (L.L.); 19954076625@163.com (H.Z.)

**Keywords:** sodium alginate, rheology, titanium dioxide nanoparticles, composite fiber, formaldehyde degradation, flame retardant

## Abstract

Because the environmental pollution arising from microplastics and carbon emissions continues to intensify, biodegradable alginate fibers have become green candidates to relieve the environmental crisis. However, the facile fabrication of alginate fibers with excellent mechanical strength and specific functionalities remains challenging. This study incorporates titanium dioxide (TiO_2_) nanoparticles into pre-crosslinked sodium alginate (SA) spinning solutions to fabricate multifunctional alginate composite fibers by a one-step wet-spinning strategy. Due to the pre-crosslinking of calcium ions (Ca^2+^), the spinning solution shows favorable rheological performance for wet spinning, ensuring the continuous fabrication of the fibers. By optimizing the TiO_2_ content, SA/TiO_2_ composite fibers exhibit oriented and uniform morphology, as well as enhanced mechanical performance (breaking stress of 400 MPa and Young’s modulus of 17.2 GPa). The incorporation of TiO_2_ also endows the fibers with excellent formaldehyde degradation and quick self-extinguished capacity, expanding their applications in formaldehyde-removal and flame-retardant textiles.

## 1. Introduction

The rapid development of the petrochemical industry has incorporated synthetic textile products into daily life, which have given rise to a series of ecological and environmental issues [1,2,3,4,5]. Because synthetic fibers are more susceptible to forming microplastics due to their high aspect ratios [6,7,8,9], fiber-based microplastics account for more than half of all microplastics in global wastewater [10]. Therefore, the development of environmentally friendly fibers has emerged as a feasible approach to address pollution challenges [11,12,13]. Sodium alginate (SA), which is extracted from algae, is a linear chain polysaccharide consisting of M (β-D-mannuronic acid) and G (α-L-guluronic acid) segments [14]. The “egg-box” structures are formed through ionic cross-linking between the G segments of SA and divalent metal ions [15,16,17], providing a feasible approach to fabricate SA fibers via wet spinning [18]. With pre-stretching, alginate fibers with a tensile strength of up to 2 cN/dtex can be obtained [19]. During the fabrication of SA fibers, the rapid cross-linking with Ca^2+^ ions creates a calcium alginate outer layer that hinders the further diffusion of Ca^2+^ ions, which leads to the formation of a skin–core structure in the resultant fibers and compromises their mechanical properties [20,21]. The relatively poor mechanical properties limit the application of alginate fibers. Thus, there are two major problems for alginate fibers: the low mechanical strength and insufficient functionalities.

The above-mentioned limitations have been addressed through various strategies, such as pre-stretching [20], adding functional additives [21,22,23,24,25], and introducing ultra-high molecular weight polymers [26]. However, these strategies for enhancing mechanical properties of alginate fibers have limitations. For example, although additives and ultra-high molecular weight polymers improve the mechanical properties of fibers, they compromise the excellent biodegradability of the fibers. The pre-stretching process only enhances fiber strength to a limited extent. Faced with these drawbacks, it is necessary to develop a novel and facile strategy to reinforce alginate fibers without compromising the biodegradability. Hao et al. pre-crosslinked SA chains with Ca^2+^ ions to regulate the rheological properties of the spinning solution and fabricated alginate multifilaments with good spinnability and breaking stress as high as 474 MPa because of the strong intermolecular hydrogen bonding and high degree of orientation [27]. This novel strategy opens a new window for the facile and continuous fabrication of alginate fibers with high strength. Titanium dioxide (TiO_2_) nanoparticles have been proven to possess the ability to decompose formaldehyde under UV light [28,29,30,31]. Thus, TiO_2_ nanoparticles can serve as functional additives to endow alginate fibers with new functionalities. However, fibers with the incorporation of TiO_2_ nanoparticles often face challenges such as uniform particle dispersion and complicated preparation processes [32,33].

In this work, Ca^2+^ pre-crosslinked SA/TiO_2_ solutions were used as spinning solutions to prepare high-strength and multifunctional composite fibers via wet spinning. The shear and extensional rheological behaviors of pre-crosslinked SA/TiO_2_ solutions with various TiO_2_ solid contents were studied. Alginate composite fibers with varying TiO_2_ mass fractions were wet spun, and the mechanical properties and morphology of the composite fibers were systematically measured. The formaldehyde degradation and flame-retardant capacity of the composite fibers with optimal TiO_2_ mass fractions were also examined.

## 2. Experimental Section

### 2.1. Materials

Sodium alginate (SA) with a viscosity of 1100 mPa·s, a G/M ratio of 2:1, and a molecular weight (Mw) of 221,500 Da was purchased from Qingdao Hyzlin Biology Development Company Limited (Qingdao, China). Ethylenediaminetetraacetic acid disodium calcium (EDTA-Ca) and glucono delta-lactone (GDL) were purchased from Aladdin Chemical Company Limited (Shanghai, China). Calcium chloride (CaCl_2_) and formaldehyde solutions (10 mg/mL) were obtained from Aladdin Chemistry Company Limited. Titanium dioxide (TiO_2_) nanoparticles with a diameter of ~200 nm were purchased from Tianjin Baima Technology Company Limited (Tianjin, China). Deionized (DI) water from Millipore (Burlington, MA, USA) was used for solution preparation. All reagents were employed as received without additional purification.

### 2.2. Preparation of SA/TiO_2_ Spinning Solution

The prescribed TiO_2_ nanoparticles were dispersed in 300 mL deionized water by ultrasonication for 1 h to prepare SA/TiO_2_ solutions with 1%, 3%, 5%, 7%, and 10% TiO_2_ relative to the mass of SA. A total of 8 g of SA powder was then added to the well-dispersed solution and mechanically stirred for 12 h until a homogeneous mixture was obtained. A total of 0.807 g of EDTA-Ca was dissolved in an appropriate amount of water, added into the SA/TiO_2_ solution, and stirred for 1 h until a uniform solution was formed to achieve the molar ratio (f) = 0.08 ([Ca^2+^]/[COO^−^ in G blocks]). Afterwards, 0.525 g of GDL was added to the spinning solution, thoroughly stirred, and left to stand in a sealed beaker at room temperature for three days to ensure the full release of Ca^2+^. Finally, a spinning solution of 2 wt% SA was obtained.

### 2.3. Preparation of SA/TiO_2_ Composite Fibers

The spinning solution was degassed by standing at room temperature and atmospheric pressure for 1 day. The spinning solution was extruded at an air pressure of 0.1 MPa, with the flow rate controlled by a gear metering pump. Before passing through a 200-mesh spinneret (orifice diameter: 100 μm), the spinning solution was first filtered through a 200-mesh metal filter to remove the impurities. The stretch ratio (SR) was set to 1 (SR = *v*_c_/*v*_e_, where *v*_c_ and *v*_e_ represent the collection speed and extrusion speed, respectively). The solution was extruded into a 5 wt% CaCl_2_ coagulation bath for crosslinking. Then, the fibers were thoroughly washed with DI water and ethanol in sequence. The sample was then left to dry at room temperature and atmospheric pressure. All spinning processes were replicated three times. The composite fiber samples for subsequent mechanical tests were randomly selected from three parallel batches.

### 2.4. Characterization

TiO_2_ was dispersed in DI water, and the resultant mixture was treated with an ultrasonic processor at 100 W and frequency of 20–25 kHz for 30 min to obtain a 0.01 wt% TiO_2_ solution. Immediately after ultrasonication, the particle size of TiO_2_ nanoparticles was measured using dynamic light scattering (DLS). X-ray diffraction (XRD) analysis of the sample was performed on an Ultima IV diffractometer. A DHR-3 rheometer from TA Instruments (New Castle, DE, USA) was used to analyze the shear rheological behaviors of SA solutions containing different TiO_2_ contents. The measurements were performed at 25 °C using a cone-plate fixture (diameter: 60 mm, cone angle: 1.5°, gap: 45 μm), after which steady shear tests were then conducted over a shear rate range of 0.01–1000 s^−1^. The non-Newtonian index can be determined by fitting the resulting data to a power-law formula:

(1) $$\eta =K\;{\overset{\cdot }{\gamma }}^{n-1}$$

where *K* is the consistency index and *n* is the non-Newtonian index, *η* is the shear viscosity, and $\overset{\cdot }{\gamma }$ is the shear rate. Extensional rheological testing of spinning solutions was performed using a CaBER 1 capillary breakup rheometer (HAAKE) that featured a 6 mm diameter fixture. The test solutions were initially gapped at 3 mm and then pre-stretched at 94 mm/s to reach a 9 mm final gap.

The Favimat-airobot single fiber testing system (Textechno, Mönchengladbach, Germany) was used to conduct mechanical tests on the fibers under certain conditions (25 ± 2 °C, 65%RH). The initial length and strain rate were set to 10 mm and 5 mm/min, respectively. The fiber diameter was measured using an optical microscope (Leica DM2700P, Leica, Wetzlar, Germany), and the cross-sectional area was calculated by the diameter. For each sample, a minimum of 10 replicate measurements were carried out. Polarized observation was carried out in transmitted light mode using a Leica DM2700P microscope (Leica, Wetzlar, Germany). A JEOL JSM-6390LV scanning electron microscope (SEM) (JEOL, Tokyo, Japan) was used to characterize the surface morphology of the composite fibers. Before SEM testing at an accelerating voltage of 15 kV, the fiber surface was sputter coated with a thin gold layer. The limiting oxygen index (LOI) of fabrics with dimensions of 150 mm × 58 mm was measured using a limiting oxygen index analyzer (TTech-GBT2406-4, TESTech, Suzhou, China).

## 3. Results and Discussion

### 3.1. Rheological Properties of SA/TiO_2_ Spinning Solutions

Large particle size and agglomeration are prone to cause nanoparticles to act as defects in the fibers, leading to stress concentration and fiber fracture under external force. Therefore, it is necessary to select TiO_2_ nanoparticles with an appropriate size that meets the requirements of fiber preparation. It can be observed that the XRD pattern of the TiO_2_ powder shows diffraction peaks near 2θ = 25°, 37°, 48°, and 55°, which are consistent with those of anatase TiO_2_ (Figure S1). As shown in Figure S2, the particle size of the employed TiO_2_ is in the range of 200–300 nm. The optical microscopy image of SA/ TiO_2_ solutions shows a small degree of aggregation of TiO_2_ nanoparticles (Figure S3).

**Figure 1:**
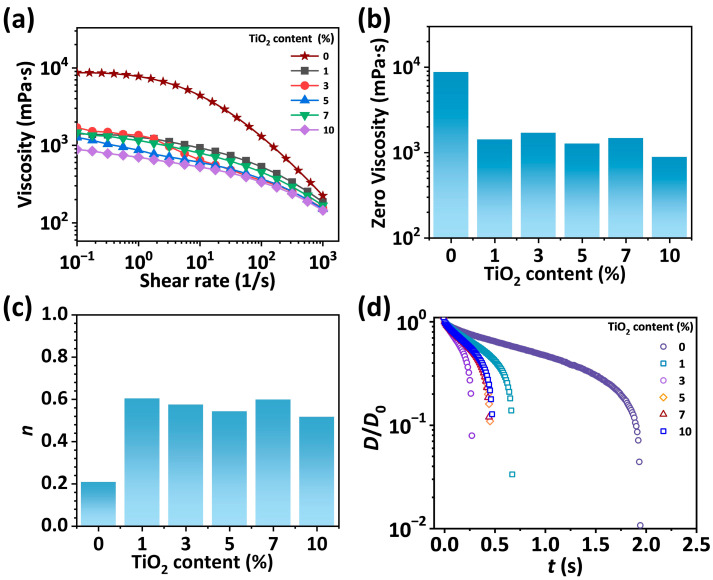
(**a**) Shear viscosity of pre-crosslinked SA/TiO_2_ solutions with *f =* 0.08 and different solid content of TiO_2_ at different shear rate. (**b**,**c**) Zero shear viscosity (**b**) and non-Newtonian index (**c**) of SA/TiO_2_ solutions. (**d**) The evolution of the normalized midpoint diameter of the SA/TiO_2_ solution filaments over time.

The shear and extensional rheological behaviors of pre-crosslinked spinning solutions (*f =* 0.08) with different TiO_2_ solid contents were analyzed to investigate their spinnability. As shown in Figure 1a, the viscosity of the solutions decreases with increasing shear rate, exhibiting a typical shear-thinning behavior. As shown in Figure 1b, the zero-viscosity of SA/TiO_2_ solutions first drastically decreases from 8000 to 1500 mPa·s with the addition of 1% TiO_2_ and then fluctuates around 1300 mPa·s with the further increase in TiO_2_ content from 1 to 10%. The drastic decrease in the viscosity by adding 1% TiO_2_ is attributed to the fact that SA chains absorb on the surface of TiO_2_ nanoparticles, which reduces the degree of entanglements of SA chains in the solutions. This phenomenon has been reported for different polymer-nanoparticle systems [34,35,36]. The fluctuation of the viscosity when TiO_2_ content is in the range of 1% to 10% is because of the interplay of two effects: the increased concentration of TiO_2_ nanoparticles and the aggregation of TiO_2_ nanoparticles that decreases the surface area for SA chain absorbing. These two effects work together to generate a viscosity without prominent variation with TiO_2_ content.

Using the five points at high shear rate in Figure 1a to perform linear fitting according to Equation (1), the non-Newtonian index (*n*) is determined by adding 1 to the slope of the fitted line (Figure 1c). When the TiO_2_ content increases from 0% to 1%, *n* increases drastically, which indicates that the introduction of TiO_2_ drastically decreases the shear-thinning degree of the solution. With TiO_2_ content increasing from 1% to 10%, the value of *n* fluctuates around 0.55, demonstrating that when the TiO_2_ content exceeds 1%, the further addition of TiO_2_ hardly affects the shear-thinning behavior of the solution. As shown in Figure 1d, after the incorporation of 1% TiO_2_, due to the reduction in the entanglement density and friction between SA chains, the pinch-off time during the capillary force-driven breakup of the SA/TiO_2_ solution decreases from 1.9 s to 0.7 s. With the further addition of TiO_2_, the pinch-off time stabilized at ~0.5 s. It was reported that the pinch-off time during the capillary force-driven breakup of SA solutions increased from 0.7 s to 1.8 s with *f* increasing from 0 to 0.08, demonstrating that pre-crosslinking with Ca^2+^ delays the relaxation of SA chains during stretching [27]. Since the pinch-off time of the solutions becomes shorter after incorporation TiO_2_, pre-crosslinking ensures sufficient pinch-off time for SA solutions containing TiO_2_ nanoparticles. This is indispensable for a good spinnability during wet spinning of the fibers.

### 3.2. Mechanical Properties of SA/TiO_2_ Composite Fibers

**Figure 2:**
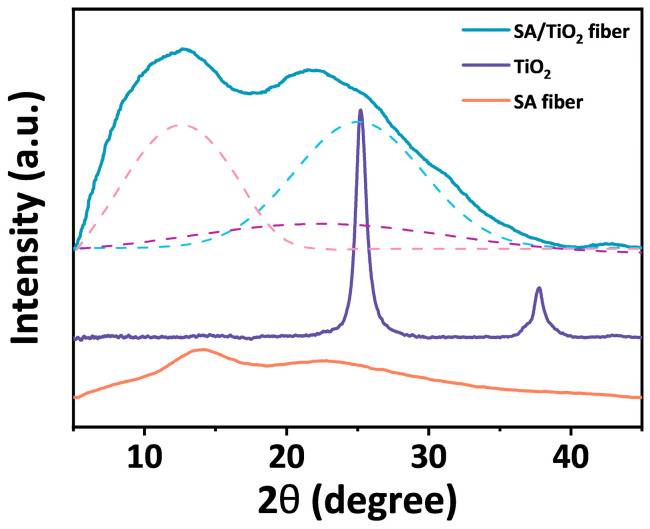
XRD patterns of TiO_2_ powder, SA fiber, and SA/TiO_2_ composite fiber. The dotted lines represent the deconvolved peaks of the pattern of SA/TiO_2_ composite fiber. The pink and purple dotted lines correspond to the characteristic peaks of SA fiber, and the blue dotted line corresponds the characteristic peak of TiO_2_.

In order to confirm whether TiO_2_ nanoparticles have been incorporated into the composite fibers, XRD tests were performed on TiO_2_, pure alginate fibers and SA/TiO_2_ composite fibers (Figure 2). Due to the low crystallinity, the pure alginate fiber exhibits broad characteristic diffraction peaks at 2θ = 13.2° and 22.5°, with the peak intensity at 22.5° lower than that at 13.2°. On the other hand, TiO_2_ exhibits a sharp diffraction peak at 2θ = 25°. Peak deconvolution was performed on the XRD pattern of SA/TiO_2_ composite fiber, and the pattern was separated into the peaks at 13.2°, 22.5°, and 25°. The diffraction peaks at 13.2° and 22.5° are consistent with the diffraction peaks of pure SA fiber, while the diffraction peak at 25° corresponds to the diffraction peak of TiO_2_. The above results confirm that TiO_2_ nanoparticles are incorporated into the alginate fibers.

**Figure 3:**
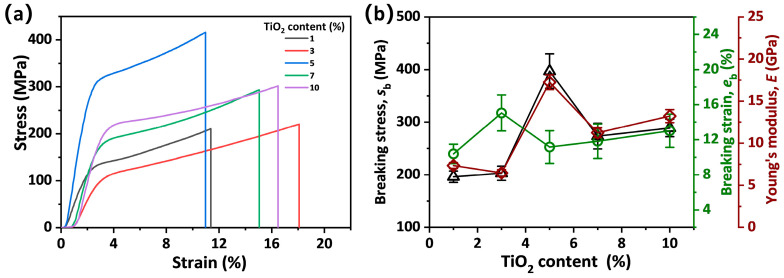
(**a**) Tensile stress–strain curves of SA/TiO_2_ composite fibers with different solid content of TiO_2_. (**b**) Mechanical parameters of SA/TiO_2_ composite fibers with different solid content of TiO_2_.

The mechanical properties of composite fibers with different TiO_2_ content were investigated. The stress-strain curves show a linear region below the strain of 2% and non-prominent yielding subsequently, followed by a strain softening region until the fibers break (Figure 3a). Figure 3b shows that the breaking stress (*s*_b_) of the composite fibers increases from 200 MPa at 1% TiO_2_ to 400 MPa at 5% TiO_2_. With a further increase in TiO_2_ content to 10%, the *s*_b_ of the fiber decreases to 289 MPa. The Young’s modulus (*E*) of the fibers also shows an increasing trend from 7.3 GPa to 17.2 GPa and then decreases to 13 GPa with the further increase in TiO_2_ content. This is because a small amount of TiO_2_ can reinforce the SA fibers due to its large specific surface area and high stiffness, whereas the excessively high content causes TiO_2_ agglomeration, which acts as defects in the fibers and hinders the effective alignment of SA chains, reducing *E* and *s*_b_. Similar phenomena are also observed in other nanoparticle-reinforced fiber materials. For example, the mechanical properties of graphene oxide-modified cellulose fibers show a trend, with tensile strength increasing and then decreasing as the graphene oxide loading increases [37]. Moreover, with the increase in TiO_2_ solid content, breaking strain (*e*_b_) shows a slight overall upward trend, increasing from 10.4% to 13%. Therefore, the spinning solution with TiO_2_ solid content of 5% is selected for the preparation of SA/TiO_2_ composite fibers. This selection is based on the rheological behavior of the spinning solutions and mechanical properties of the fibers.

### 3.3. Morphology of SA/TiO_2_ Composite Fibers

**Figure 4:**
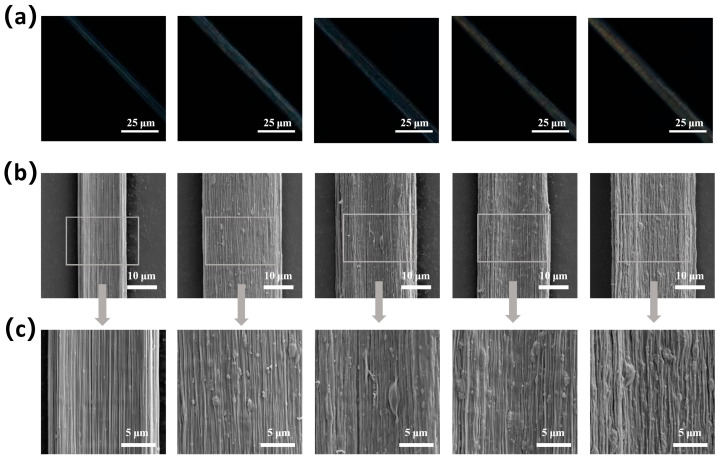
(**a**) Polarized optical microscopy images of SA/TiO_2_ composite fibers with TiO_2_ solid content of 1%, 3%, 5%, 7%, and 10% from left to right, respectively. (**b**) SEM images of SA/TiO_2_ composite fibers with TiO_2_ solid contents of 1%, 3%, 5%, 7%, and 10% from left to right, respectively. (**c**) High-magnification SEM images of the enclosed areas in (**b**).

Figure S4 shows the photograph of continuously fabricated composite multifilaments with a TiO_2_ solid content of 5% after drying and collection. SA/TiO_2_ fibers exhibit an overall milky white appearance due to the incorporation of TiO_2_. The polarized micrograph of the composite fibers with different TiO_2_ contents shows a white birefringent color (Figure 4a). This is because SA chains in the fibers were oriented along the fiber axis due to the confined radial shrinkage. The composite fibers exhibit diameters ranging from 10 to 20 μm. Figure 4b,c are SEM of composite fibers, where microfibrillar structures distributed along the radial direction can be observed. Meanwhile, it can be clearly seen that the number of particles attached to the fiber surface increases significantly with the increase in TiO_2_ content. Notably, obvious particle aggregation of TiO_2_ appears on the surface of fibers with high TiO_2_ contents of 7% and 10%. This confirms that the decrease in tensile strength of composite fibers with high TiO_2_ content is caused by the agglomeration of nanoparticles. The elemental distribution of titanium on the composite fiber surface is presented through EDS images. As shown in Figure S5 and Table S1, with the increase in TiO_2_ content, the number of green bright spots representing the titanium element becomes larger. However, aggregation of titanium element can be observed in the images of composite fibers with high TiO_2_ content, which is consistent with the SEM observation. It should be noted that the content of titanium element obtained from EDS is lower than that in the spinning solutions. This is because EDS only analyzes the surface of the fiber. Moreover, during fiber formation, TiO_2_ nanoparticles may diffuse into the coagulation bath, which also reduces the TiO_2_ content.

### 3.4. Formaldehyde Degradation and Flame-Retardant Properties of SA/TiO_2_ Composite Fibers

**Figure 5:**
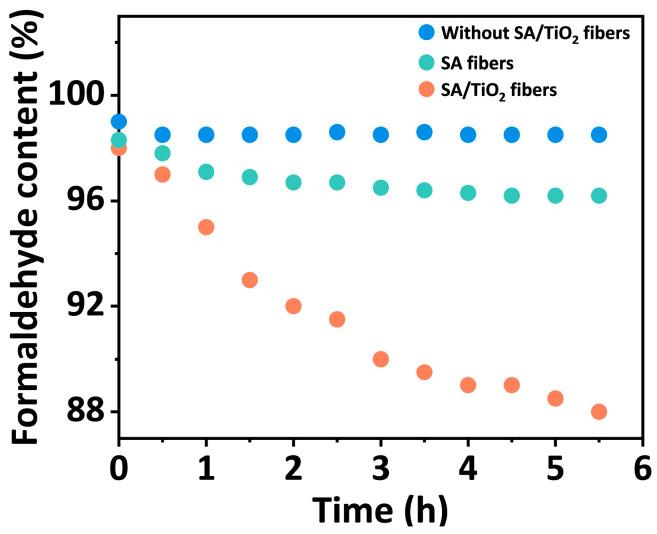
Comparison of the relative formaldehyde content over time without fibers, after placing SA fibers, and after placing SA/TiO_2_ composite fibers with TiO_2_ solid contents of 5%.

A self-made device was used to test the formaldehyde purification capacity of SA/TiO_2_ composite fibers (Figure S6). The airtightness of the device was ensured before the experiment. The test was conducted under daylight, utilizing the ultraviolet spectrum in the daylight to achieve photocatalytic degradation of formaldehyde. A total of 50 μL of formaldehyde standard solution was diluted to 1% and then added dropwise into a small petri dish using a pipette. After the solution was completely volatilized, the concentration change was continuously monitored by an air quality monitor. The measured concentrations were then normalized with the initial concentration to calculate the variation rate of formaldehyde content. The blue data points in Figure 5 represent the variation of formaldehyde concentration in the device without composite fibers. No obvious change in concentration is observed within 6 h. The green data points in Figure 5 represent the changes in formaldehyde concentration in the presence of pure SA fibers. Within 6 h, the formaldehyde concentration decreases from 98% to 96% due to the physical adsorption of formaldehyde by the fibers. By placing 5 g of SA/TiO_2_ composite fibers with 5% TiO_2_ solid content in the glass petri dish at the center of the test chamber, a distinct downward trend in the formaldehyde concentration within the test chamber was observed. After 6 h, the concentration decreases to approximately 90% of its initial value (orange data points in Figure 5).

**Figure 6:**
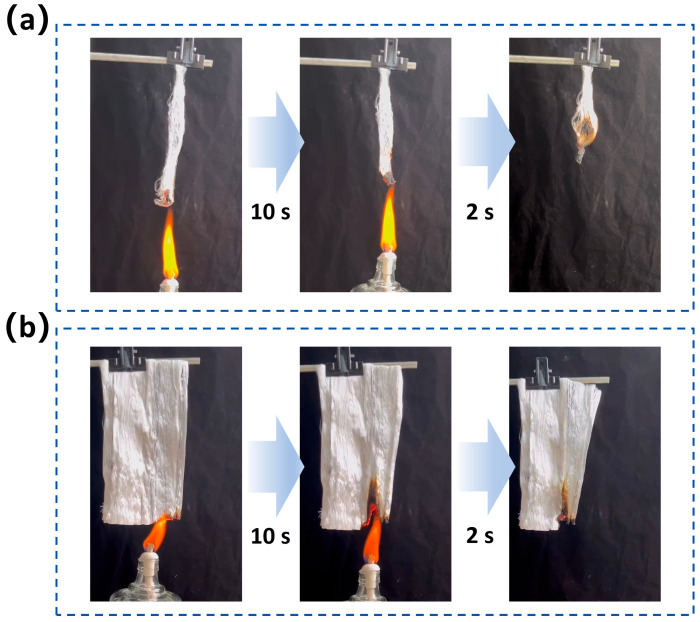
Flame-retardant testing of SA/TiO_2_ composite fibers (**a**) and fabrics (**b**) with TiO_2_ solid contents of 5%.

Because Ca^2+^ crosslinked SA and TiO_2_ nanoparticles both possess flame retardant properties [38,39,40,41,42], the SA/TiO_2_ composite fibers also have great potential in flame retardant fabrics. Flame retardant tests were conducted on the SA/TiO_2_ composite fibers (5% TiO_2_ content) and fabrics. The samples were suspended above an alcohol lamp flame for combustion observation. As shown in Figure 6a, after 10 s of exposure to the flame, no open flame was observed on the fibers, and blackish-gray ash formed in the burned area. After removing the fire source, the fiber bundle self-extinguished within 2 s. The SA/TiO_2_ composite fabric also exhibits excellent flame-retardant performance. A vertical combustion test was performed on an SA/TiO_2_ composite fabric with a size of approximately 15 cm × 10 cm (Figure 6b). After about 10 s of ignition, the fabric still maintained the structure after removing the alcohol lamp, and no open flame was observed. Moreover, the average LOI test of the composite fabrics is approximately 35%, confirming the flame-retardant properties of the composite fibers. The above observations demonstrate that SA/TiO_2_ composite fibers have excellent formaldehyde degradation and flame retardant capacity.

## 4. Conclusions

In summary, multifunctional alginate-based composite fibers are successfully designed and prepared in this study. TiO_2_ nanoparticles are incorporated into pre-crosslinked SA spinning solutions, which are subsequently used for the continuous fabrication of SA/TiO_2_ composite fibers via wet spinning. The shear and extensional rheological behaviors of pre-crosslinked SA/TiO_2_ solutions are systematically examined. It is shown that the pre-crosslinked SA/TiO_2_ solutions with TiO_2_ content of 5–10% exhibit a zero-viscosity of ~1300 mPa·s and pinch-off time of 0.5 s, ensuring a favorable spinnability of the fibers. The oriented and aligned microstructures of the alginate matrix and the dispersed TiO_2_ nanoparticles strengthen the mechanical performances of composite fibers. When the TiO_2_ content reaches 5%, the composite fibers exhibit optimal mechanical properties with a breaking stress of 400 MPa, breaking strain of 11%, and Young’s modulus of 17.2 GPa. The incorporation of TiO_2_ nanoparticles also endows the fibers with good formaldehyde degradation and flame-retardant capacity, demonstrating the potential applications of alginate fibers in formaldehyde removal and protective textiles.

## Data Availability

The original contributions presented in this study are included in the article/Supplementary Material. Further inquiries can be directed to the corresponding author.

## Supplementary Materials

The following supporting information can be downloaded at: https://www.mdpi.com/article/10.3390/ma19101933/s1, Figure S1: Comparison of the XRD pattern of TiO_2_ used in this experiment with the standard XRD card of anatase TiO_2_. Figure S2: The size distribution of TiO_2_ nanoparticles. Figure S3: Optical microscopy images of pure SA solution (a) and SA/TiO_2_ solution with TiO_2_ content of 5% (b). Figure S4: Photo of SA/TiO_2_ composite fibers. Figure S5. EDS mapping images of titanium element distribution on the surface of SA/TiO_2_ composite fibers. Figure S6: Self-made formaldehyde removal rate testing device. Table S1: Titanium element content in SA/TiO_2_ composite fibers characterized by EDS.
