# Supplementary material for: Multifunctional Alginate Composite Fibers Based on Pre-Crosslinked Spinning Solutions
Source: Materials (Basel). 2026 May 8;19(10):1933. doi: 10.3390/ma19101933 (PMC13208577; doi:10.3390/ma19101933)
Supplement: Supplementary file 1 [file materials-19-01933-s001.zip › materials-4286367-supplementary.pdf]

Supporting Information

Multifunctional Alginate Composite Fibers Based on Pre-Crosslinked Spinning Solutions

Lingchun Liu†, Hanxu Zhou†, Cong Du*

Shandong Key Laboratory of Renewable Membrane Materials, College of Materials Science and Engineering, Qingdao University, Qingdao 266071, China

†These authors contributed equally to this work.

*Corresponding authors. *E-mail addresses*: cong.du@qdu.edu.cn (C. Du)

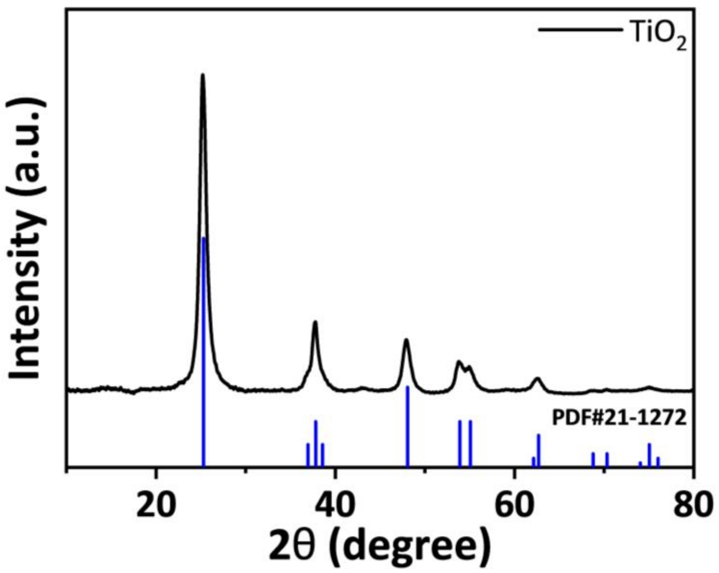

Figure S1. Comparison of the XRD pattern of TiO_2 used in this experiment with the standard XRD card of anatase TiO_2.

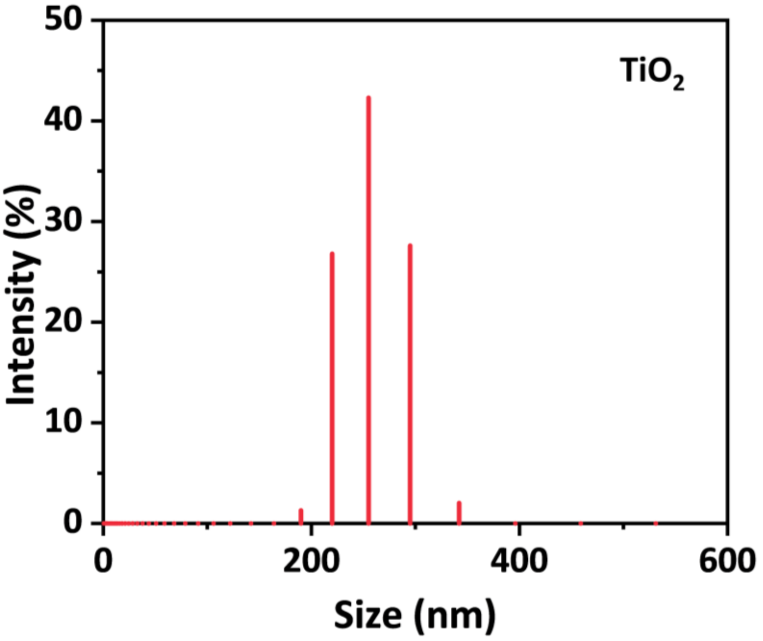

Figure S2. The size distribution of TiO_2 nanoparticles.

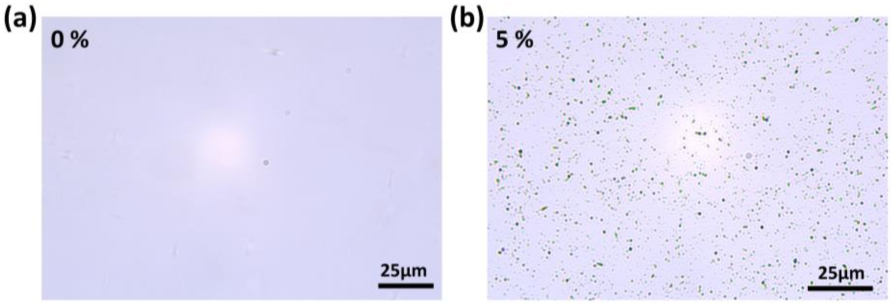

Figure S3. Optical microscopy images of pure SA solution (a) and SA/TiO_2 solution with TiO_2 content of 5% (b).

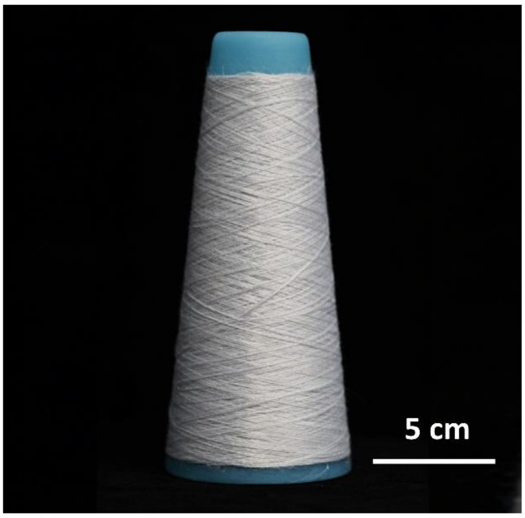

Figure S4. Photo of SA/TiO_2 composite fibers.

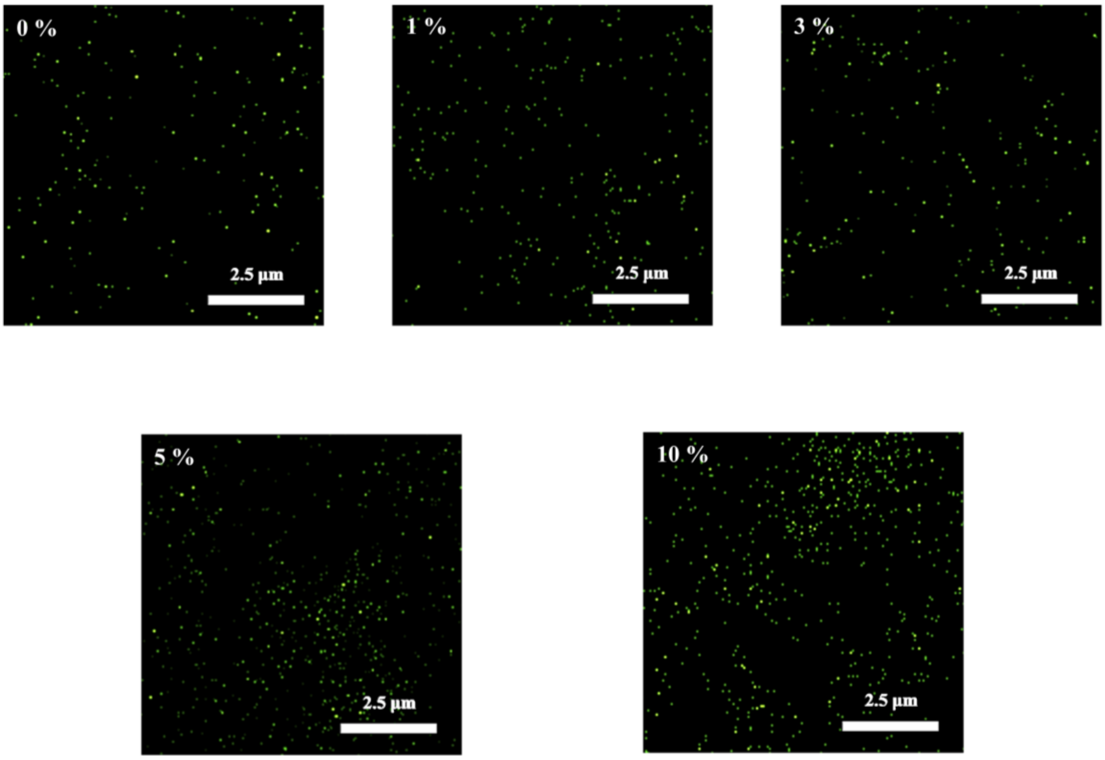

Figure S5. EDS mapping images of titanium element distribution on the surface of SA/TiO_2 composite fibers.

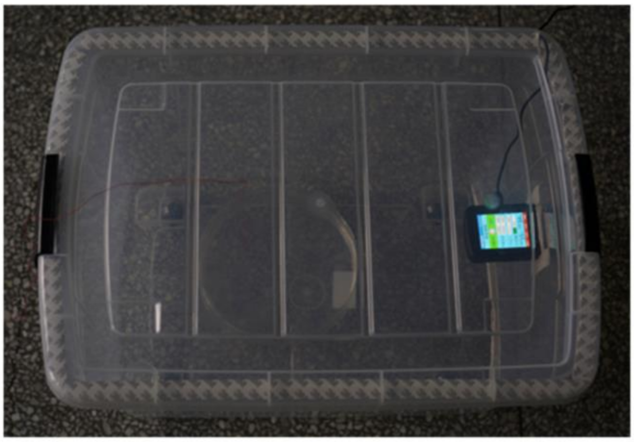

Figure S6. Self-made formaldehyde removal rate testing device.

Table S1 Titanium element content in SA/TiO_2 composite fibers characterized by EDS.

**TiO_2 content (wt%)**	1	3	5	7	10
**Ti content (Atomic %)**	1.41	1.69	2.39	4.31	4.38
